# Stress-diathesis based predictors of depression and anxiety trajectories in adolescence: a population-based longitudinal cohort study

**DOI:** 10.1017/S0033291726103560

**Published:** 2026-03-03

**Authors:** Philip J. Batterham, Kate Maston, Bridianne O’Dea, Lyndsay Brown, Alison L. Calear, Mark Larsen, S. Rachel Skinner, Helen Christensen, Aliza Werner-Seidler

**Affiliations:** 1 Centre for Mental Health Research, Australian National University, Canberra, Australia; 2 Black Dog Institute, University of New South Wales, Sydney, Australia; 3 Flinders Institute for Mental Health and Wellbeing, Flinders University, Adelaide, Australia; 4 Centre for Big Data Research in Health, University of New South Wales, Australia; 5 Speciality of Child and Adolescent Health, The University of Sydney, Sydney, Australia; 6 School of Psychiatry, University of New South Wales, Australia

**Keywords:** adolescent mental health, anxiety, depression, prevention, trajectories

## Abstract

**Background:**

Adolescent mental health has worsened, and prevention efforts have become increasingly important. The purpose of this study was to examine longitudinal symptom trajectories of depression and anxiety throughout adolescence, in a contemporary sample. The stress–diathesis model was used to inform potential vulnerability factors and stressors associated with these trajectories.

**Methods:**

Symptoms of depression and generalized anxiety were assessed in a school-based population sample of *N* = 6102 adolescents (aged 13–14 at baseline). Growth mixture models across four time points were used to model longitudinal trajectories of symptoms. Multinomial regression was used to examine factors associated with each trajectory class.

**Results:**

Of the full sample, 49.5% were female, 45.9% were male, and 4.6% were gender diverse. Four discrete classes for both depression and anxiety trajectories were identified, which comprised consistently low symptoms (‘low’; 72.5% depression; 66.9% anxiety), consistently high symptoms (‘high’; 11.5% depression; 18.4% anxiety), elevated symptoms that reduced over time (‘decreasing’; 8.3% depression; 6.9% anxiety), and low-moderate symptoms that increased over time (‘increasing’; 7.7% depression; 7.8% anxiety). Factors associated with poorer trajectories were being female or gender diverse, lower socioeconomic status, higher levels of neuroticism and lower levels of conscientiousness, greater adverse childhood experiences, higher levels of peer problems, bullying victimization, and negative family interactions.

**Conclusions:**

A range of background vulnerabilities and specific stressors were associated with poorer depression and anxiety trajectories over a 3-year period. Prevention approaches may require policy and practice changes that promote more supportive family, school, and societal environments from childhood to adolescence.

The prevalence of depression and anxiety tends to increase markedly throughout adolescence. Approximately half of mental health conditions emerge before the age of 18 years, with anxiety disorders often having a median onset during adolescence, and depression during early adulthood (Kessler et al., 2005; Solmi et al., 2022). Early identification of young people most at risk of these disorders presents an opportunity to deliver interventions that may circumvent disorder onset. Furthermore, identifying the risk factors that contribute to poorer trajectories of mental health may be used to produce more effective and tailored prevention approaches. Trajectory-based models may reveal the factors associated with symptom levels and change for an individual, or identify etiologically distinct groups for whom tailored therapeutic interventions can be developed. Focusing on group-level trajectories may be important for characterizing the prevalence of mental health risk, understanding etiology, and better tailoring and targeting of interventions. Many existing prevention programs are delivered universally to all young people without tailoring and have only modest effects (Hayes et al., 2025; Werner-Seidler et al., 2021). Targeting interventions to young people who are most at risk based on characteristics associated with poorer outcomes may lead to more efficient and effective approaches.

Identifying developmental trajectories of depressive and anxiety symptoms during adolescence involves recognizing an interaction between biological, psychological, and/or environmental factors that converge at different developmental stages, shaping divergent pathways for young people (Daskalakis et al., 2013). Accordingly, employing a theoretical framework to conceptualize the development of these trajectories provides a useful lens for examining how and why different factors combine to produce varying trajectories (Sterba & Bauer, 2010). The stress–diathesis model proposes that psychological symptoms arise from the interaction between one’s vulnerability (diathesis) and external stressors (Monroe & Simons, 1991). Accordingly, many factors shaping adolescent trajectories of mental health may be characterized as either background vulnerabilities (diatheses) or as stressors experienced during adolescence. For example, vulnerability factors may involve biological (such as genetic factors), psychological (such as temperament), or environmental (such as child maltreatment) factors (Humphreys et al., 2020; Prince et al., 2021; Wilde et al., 2014). According to the model, the presence of vulnerability factors amplifies the risk that external stressors will interact with these pre-existing vulnerability factors to increase the risk for depression (Ingram & Price, 2001). Empirical studies directly testing the stress–diathesis hypothesis have consistently provided evidence supporting this model as a valid framework for understanding the development of depression and anxiety disorders (e.g. Brozina & Abela, 2006; Colodro-Conde et al., 2018; Ingram & Price, 2001; Kendler, Myers, & Prescott, 2002; Reinelt et al., 2013). Accordingly, we used this model to guide the selection of variables expected to impact developmental trajectories of depression and anxiety across stress and diathesis domains.

The last two decades have seen an emergence of studies examining longitudinal trajectories of adolescent depression and anxiety. A systematic review identified 13 studies that had examined longitudinal trajectories of depressive symptoms in adolescents, and found evidence of subgroups with differing trajectories, which were associated with poorer outcomes in adulthood (Dekker et al., 2007; Shore, Toumbourou, Lewis, & Kremer, 2018). Together, these studies have identified between three and six distinct trajectory classes, with the largest class following a ‘no’ or ‘low’ symptom trajectory, and smaller proportions experiencing ‘high’, ‘increasing’, or ‘decreasing’ depression symptom trajectory (Shore et al., 2018). Variation across the literature in class number likely reflects differences in the tools used to measure depression. Shore and colleagues found that gender, socioeconomic status, conduct problems, and stressful life events were associated with higher risk depression trajectories, although there were some inconsistencies across studies (Shore et al., 2018).

Fewer studies have examined developmental trajectories of anxiety, producing mixed results in relation to the number of distinct trajectories identified, ranging from two to five different trajectories (e.g. Crocetti et al., 2009; Morin et al., 2011;Salto et al., 2025 ; Spence, Lawrence, & Zubrick, 2022). Several of the more recent studies (Salto et al., 2025; Spence et al., 2022) each identified three distinct classes, involving a ‘low’, ‘low-increasing’, and ‘high-decreasing’ trajectory. The variation in these findings likely reflects differences in methodologies and statistical approaches used to identify classes. The predictors of class membership that have been explored have also varied across studies and have been limited in terms of the breadth of predictors examined. For example, one study found social support from parents and teachers and peer victimization predicted a ‘low-increasing’ trajectory (Spence et al., 2022), while another study found higher intelligence predicted a ‘low-increasing’ trajectory (Salto et al., 2025). Mirroring the depression literature, female gender has been consistently associated with a higher risk of anxiety symptom trajectory (Crocetti et al., 2009; de la Torre-Luque et al., 2020; Salto et al., 2025).

Although data-driven, longitudinal, trajectory-based approaches are becoming more common, the existing literature is limited in several ways. First, there is a lack of convergence of the distinct trajectories of depression and anxiety during adolescence. Second, there is very limited knowledge about anxiety trajectories. Third, there is significant variation in the predictors of trajectories that have been explored, which most often do not take a theory-informed approach. Fourth, studies seldom involve investigations of both depression and anxiety trajectories within the same cohort. This underscores the need for longitudinal cohort studies of large representative samples of young people that include theoretically informed predictors of trajectories.

The present study had two aims. The first aim was to identify distinct trajectories of symptoms of depression and generalized anxiety among a large population-based cohort of adolescents who were aged approximately 14 years at the time of first assessment (baseline; Year 8) and followed up for 3 years (12, 24, and 36 months, until students were in Year 11). In line with evidence suggesting that depression and generalized anxiety follow different developmental trajectories during adolescence (Van Oort et al., 2009), we examined these illness trajectories separately. The second aim was to identify factors associated with poorer symptom trajectories, framed around the stress–diathesis model to better understand potential pathways to mental distress.

## Methods

### Participants and procedures

This study used data from the Future Proofing Study, a longitudinal cohort of Australian adolescents who were in Year 8 (*M*_age_ = 13.91, SD = 0.57) at baseline. Data from follow-up (12, 24, and 36 months) assessments were used to track trajectories of depression and generalized anxiety symptoms. The study was registered (ACTRN12619000855123), a prospective protocol was published (Werner-Seidler et al., 2020), and the study received ethical approval from the UNSW Human Research Ethics Committee (HC180836), NSW Government State Education Research Applications Process (SERAP 2019201), and relevant Catholic Schools Dioceses. A randomized controlled trial of the SPARX program, a digital cognitive behavioural therapy game, was embedded within the study (Werner-Seidler et al., 2025). The intervention had no significant effects, and there was no univariate association between trial condition and either depression trajectory (*χ^2^* = 1.69, *p* = 0.64) or anxiety trajectory (*χ^2^* = 5.60, *p* = 0.13). Consequently, both trial conditions were included in the models, and no further adjustment was made for the trial condition.

Participants were recruited from 134 high schools in Australia, with 85.6% of students attending school in the state of NSW. Most students (86%) attended schools in major cities, while 24% attended schools in regional areas, reflecting the distribution of the Australian population (AIHW, 2021). Approximately half (50.9%) of students attended government secondary schools (the remainder being non-government schools), which is slightly lower than the proportion of students attending government schools in the population (ACARA, 2021). Participants attended schools with a median index of socioeconomic advantage of 1048, which is slightly higher than the Australian population median of 1000 (ACARA, 2020). Cohort demographics at baseline were broadly representative of the Australian adolescent population (Werner-Seidler et al., 2023).

Parents and students provided informed consent before students completed questionnaires at in-school sessions facilitated by the research team. Students accessed the confidential questionnaires via a secure online portal and completed these digitally in 45-minute sessions. Participants were eligible for inclusion if they had complete data for depression and/or anxiety symptoms for at least one of the four assessments. Overall, 6102 (96%) adolescents were included in the study, with 76% completing the 12-month assessment, 65% completing the 24-month assessment, and 55% completing the 36-month assessment.

### Measures: outcomes

#### Depressive symptoms

The Patient Health Questionnaire Adolescent Version (PHQ-A) is a nine-item depression severity screening tool that aligns with diagnostic criteria from the DSM-IV and has been adapted from the PHQ-9 for adolescents (Johnson, Harris, Spitzer, & Williams, 2002; Kroenke, Spitzer, & Williams, 2001; Nandakumar et al., 2019). Higher scores indicate greater symptoms. Total scores of 5, 10, 15, and 20 represent thresholds for mild, moderate, moderately severe, and severe depression, respectively. In the current study, the internal consistency of the PHQ-A was high (*α* = 0.88).

#### Anxiety symptoms

The six-item generalized anxiety subscale of the Spence Children’s Anxiety Scale was used (SCAS; Spence, 1998). Greater scores indicate greater anxiety, and there are no universal clinical threshold scores available. In the present study, the internal consistency of the SCAS (generalized anxiety subscale) was high (*α* = 0.88).

### Measures: demographics

#### Age

Participants reported their date of birth at baseline, which was used to calculate age at each assessment point.

#### Gender

Participants reported current gender identity with options: male, female, non-binary, other, and prefer not to say. Responses were categorized into ‘male’, ‘female’, and ‘gender diverse’ (non-binary, other, and prefer not to say).

#### Language spoken

Linguistic diversity was assessed using a single item (‘What language do you speak most at home?’). Due to low numbers of participants reporting individual languages other than English, responses were dichotomized into ‘English speaking family’ and ‘non-English speaking family’.

### Measures: diatheses

#### Index of Community Socio-Educational Advantage (ICSEA)

Participants’ school-level socio-educational advantage was assessed through the ICSEA (Australian Curriculum, 2020). ICSEA is an Australian metric developed by the Australian Curriculum, Assessment and Reporting Authority (ACARA) to reflect the level of socio-educational advantage of students at a school. It is based on factors such as parents’ occupation and education levels, the school’s geographical location, and the proportion of Indigenous students. The national median ICSEA value is 1000, with higher scores indicating greater advantage.

#### Index of Relative Socio-Economic Disadvantage (IRSD) Decile

Participants’ area-level socioeconomic status was categorized using deciles of the IRSD (Australian Bureau of Statistics, 2023b) based on the postcode of residence recorded at the 2021 Australian Census (Australian Bureau of Statistics, 2023c). The IRSD, developed by the Australian Bureau of Statistics, is a commonly used measure that combines a range of census-collected variables that reflect disadvantage, including income, education level, employment status, occupation, and housing characteristics. Areas are ranked on a decile scale from 1 (relatively greater disadvantage) to 10 (relative lack of disadvantage).

#### Personality

The Big Five Personality Inventory (BFI-10; Rammstedt & John, 2007) is a 10-item index measuring the Big Five personality traits: Extraversion, Agreeableness, Conscientiousness, Neuroticism, and Openness. Respondents indicate their level of agreement with each trait on a 5-point scale, ranging from 1 (disagree strongly) to 5 (agree strongly). Total subscale scores are calculated by summing item scores (two items per subscale), ranging from 2 to 10. The BFI-10 has demonstrated acceptable test–retest reliability and good construct validity across studies (Balgiu, 2018; Rammstedt & John, 2007).

#### Adverse childhood events (ACEs)

ACEs were assessed using a modified version of the Behavioral Risk Factor Surveillance System–Adverse Childhood Experience (BRFSS-ACE), developed by the US Centers for Disease Control and Prevention (Centers for Disease Control and Prevention (CDC), 2009). The modified version of the BRFSS-ACE included 6 items from the original measure: household mental illness; household alcohol use; household illegal street drug use or prescriptive drug abuse; household incarceration; parental separation or divorce; and being sworn at, insulted, or put down by a parent or adult in the home. Two additional items assessed out-of-home care or foster care, and feelings of endangerment or physical harm. The total number of ACEs endorsed was summed.

#### Disability

Disability diagnoses were assessed by asking participants to report whether they had ever been diagnosed with: autism or Asperger’s syndrome; an intellectual disability; a specific learning disability; Tourette syndrome or other chronic tic disorder; cerebral palsy; acquired brain injury; other neurological disability; hearing impairment; or visual impairment. Due to low endorsement of individual diagnoses, and to maintain sufficient statistical power, responses were dichotomized into ‘≥1 disability diagnoses’ or ‘no disability diagnoses’.

### Measures: stressors

#### Peer relationship problems

The Strengths and Difficulties Questionnaire (SDQ) is a 25-item instrument composed of five subscales, each with five items (Goodman, 1997; Vostanis, 2006). The peer relationship problems subscale was included in this study, with scores ranging from 0 to 10, with higher scores indicating greater levels of difficulty. Internal consistency was low-moderate (*α* = 0.57).

#### Bullied

Two items assessed how frequently participants had been bullied and cyberbullied in the previous 12 months, with responses combined to form an index ranging from 2 to 10, with scores of 2 indicating no bullying and higher scores indicating greater levels of bullying victimization. These questions were adapted from The Sources of Strength Australia Project (Calear et al., 2016), which was originally derived from elsewhere (Klomek et al., 2007).

#### Negative family and friend interactions

Negative family and friend interactions were measured using an abbreviated version of the Schuster Social Support Scale (Schuster, Kessler, & Aseltine, 1990). Three items measured negative interactions with family, and three items measured negative interactions with friends. Items were rated on a 4-point Likert scale (1 = never to 4 = often). Subscale scores for negative family interactions and negative friend interactions were calculated by averaging the relevant items. Higher scores indicated greater levels of demands, criticism, and arguments in family and friend interactions. Internal consistency was high for the family subscale (*α* = 0.83) and the friends subscale (*α* = 0.76).

### Analyses

Of the total number of 6388 participants in the Future Proofing Study, 6102 (96%) had complete data for independent and dependent variables and were included in the analyses. To account for multiple comparisons, the Type I error rate (alpha) was conservatively set at 0.01 for all analyses.

Trajectories of depression and anxiety symptoms were characterized using growth mixture models (GMM) estimated across the four data points assessed annually (baseline, 12, 24, and 36 months) using all available data. GMM is used to distinguish heterogeneous latent classes of individuals with different longitudinal trajectories, based upon a set of continuous variables measured longitudinally that include all available data under the missing-at-random assumption (Muthén & Muthén, 2000). GMM combines growth modelling with latent class analysis to classify a sample with longitudinal observations into a discrete number of latent classes. The optimal number of latent classes was selected based on the significance of the Bootstrapped Likelihood Ratio Test (BLRT), testing whether a model with *k* latent classes fits significantly better than a model with one fewer (*k*–1) class (McLachlan & Peel, 2000; Nylund, Asparouhov, & Muthén, 2007), and whether the smallest class in the model represents ≥5% of the total sample, as smaller classes are hard to identify and may be unstable (Nylund et al., 2007). The interpretability or distinctiveness of the trajectories was also considered, along with other fit indices including the Akaike Information Criteria (AIC) and Bayesian Information Criterion (BIC), and entropy as an indicator of classification precision (Nylund et al., 2007). The model selection process was conducted independently for depression symptom trajectories and anxiety symptom trajectories, with models having two to seven classes examined as candidates for each outcome. GMM models included terms for intercept, linear change, and quadratic change. The characteristics of trajectories in the final model were examined descriptively, and concordance between depression and anxiety trajectory classes was determined based on crosstabulation and Spearman’s correlation.

To examine whether stress–diathesis factors were associated with each trajectory, multinomial logistic regression analyses were estimated, with odds ratios reported based on original units. The reference category for these analyses was set as the group with the lowest symptoms, with post-hoc comparisons also conducted to compare any additional pairs of trajectories that started with similar intercepts before diverging. There was evidence of negligible levels of multicollinearity among the independent variables, with all variance inflation factor estimates <2.

Two forms of sensitivity analyses were conducted. Firstly, potential clustering effects of the school were accounted for in analogous regression analyses using generalized mixed effects modelling that included a random intercept for the school. Secondly, multiple imputation was used to test whether missing data on independent variables influenced the observed associations. Five missing data imputations were produced using the Markov chain Monte-Carlo method using fully conditional specification with Gibbs sampling for the variables that were included in the regression analyses. Factors significantly associated with missingness at any follow-up included higher baseline depression scores (*t* = −3.94, *p* < .001), greater socioeconomic disadvantage based on ICSEA (*t* = 9.07, *p* < .001) or IRSD (*t* = 2.55, *p* = .005), lower agreeableness (*t* = 3.19, *p* < .001), less extroversion (*t* = −3.63, *p* < .001), greater peer problems (*t* = −5.23, *p* < .001), bullying (*t* = −7.04, *p* < .001), adverse childhood events (*t* = −6.57, *p* < .001), negative friend interactions (*t* = −3.45, *p* < .001), and male gender (*χ*^2^ = 71.9, *p* < .001), although associations with attrition were all small (Cohen’s *d* < 0.22).

## Results

The total sample was *N* = 6102 students, and the sample characteristics for continuous variables are provided in Table 1, with zero-order correlations for depression and anxiety measures in Supplementary Table S1. Nearly half of the participants identified as female (*n* = 3020, 49.5%), 2800 as male (45.9%), and 282 (4.6%) as gender diverse. Participants who spoke a non-English language at home represented 6.4% of the sample (*n* = 393), and 12.4% (*n* = 756) reported one or more disabilities. Socioeconomic indices both reflected slightly above average levels of advantage, as the national mean for ICSEA is 1000 (higher = more advantaged), and IRSD deciles above 5 indicate relatively lower levels of social disadvantage. Participants reported an average of 1.4 adverse childhood experiences, low levels of bullying (*M* = 2.9 from a possible range 2–10), low levels of peer problems (*M* = 2.3 from a possible range 0–10), and low-moderate levels of negative interactions with friends and family (*M* = 2.0 and 2.3 respectively from a range 1–4). Personality indices demonstrated moderate levels of agreeableness, conscientiousness, extroversion, neuroticism, and openness to experience.

**Table 1.** Characteristics of the sample (*n* = 6102)

	Mean	SD
PHQ-A depression score – baseline (*n* = 6102)	7.35	6.29
PHQ-A depression score – 12 months (*n* = 4658)	7.25	6.16
PHQ-A depression score – 24 months (*n* = 3992)	7.34	6.04
PHQ-A depression score – 36 months (*n* = 3360)	7.20	5.72
SCAS generalized anxiety score – baseline (*n* = 6102)	6.54	4.49
SCAS generalized anxiety score – 12 months (*n* = 4639)	6.28	4.51
SCAS generalized anxiety score – 24 months (*n* = 3978)	6.30	4.49
SCAS generalized anxiety score – 36 months (*n* = 3339)	6.19	4.35
Age in years – baseline (Year 8)	13.91	0.57
ICSEA socioeconomic status	1048.70	79.75
IRSD decile	6.67	2.78
Agreeableness	7.45	1.63
Conscientiousness	6.28	1.81
Extroversion	6.57	2.08
Neuroticism	6.12	2.13
Openness to experience	6.59	1.51
Adverse childhood events	1.36	1.42
Peer problems	2.33	1.86
Bullied	2.90	1.50
Negative friend interactions	2.00	0.71
Negative family interactions	2.28	0.84

*Note*: PHQ-A, Patient Health Questionnaire-Adolescent Version; SCAS, Spence Child Anxiety Scale; ICSEA, Index of Community Socio-Educational Advantage; IRSD, Index of Relative Socio-economic Disadvantage.

**Figure 1.**
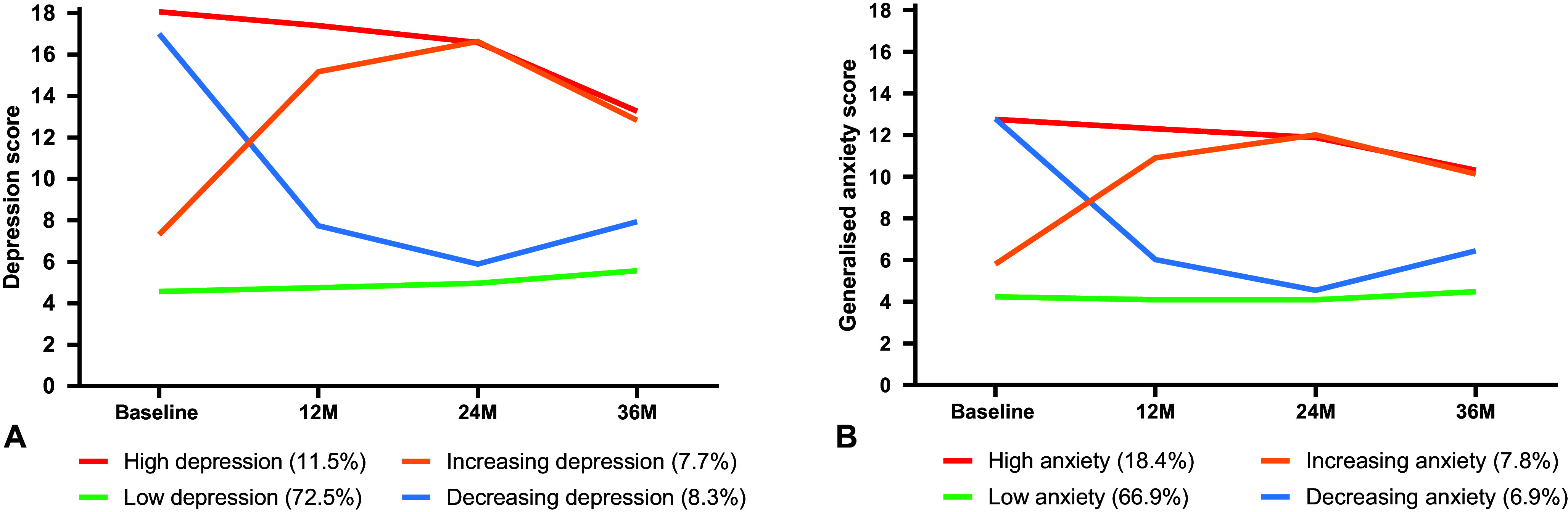
Panel A shows raw means of depression symptom scores (Patient Health Questionnaire-Adolescent) across the follow-up period for the four classes of depression trajectories (range 0–27); Panel B shows raw means of anxiety symptom scores (Spence Child Anxiety Scale-Generalized Anxiety subscale) across the follow-up period for the four classes of anxiety trajectories (range 0–18).

Table 2 compares the candidate GMM models for symptoms of depression and generalized anxiety. In both cases, the BLRT was significant for increasing the number of classes in all models. Therefore, class size, distinctiveness of trajectories, and relative change in AIC, BIC, entropy, and interpretability of trajectories were used to select the final number of classes. For both symptom outcomes, the four-class models had optimal features across these criteria and were subsequently selected. The depression and anxiety classes (Figure 1) had similar features – in both cases, there was a class with consistently low symptoms (‘low’), one with consistently high symptoms (‘high’), one with high symptoms that reduced over time (‘decreasing’), and one with low-moderate symptoms that increased over time (‘increasing’). There was some overlap between depression versus anxiety classes, with a Spearman’s correlation of 0.49 suggesting moderate differentiation between depression and anxiety symptom trajectories. For both depression and anxiety, the majority of participants were in the low symptom class. The low depression class represented 72.5% of the sample, with the high class comprising 11.5%, increasing 7.7%, and decreasing 8.3%. For anxiety, the low class comprised 66.9% of the sample, with 18.4% in the high symptom class, 7.8% increasing, and 6.9% decreasing. Estimates of intercept, linear change and quadratic change from the GMMs are summarized in Supplementary Table S1, indicating that both low and high depression classes had no significant change over time, the decreasing and increasing depression and anxiety classes had significant linear and quadratic changes, the high anxiety class had no significant change, and the low anxiety class had a small linear decrease in conjunction with a small quadratic increase in symptoms.

**Table 2.** Comparison of candidate growth mixture models

*k*_classes_ in model	Two classes	Three classes	Four classes	Five classes	Six classes	Seven classes
**Depression models**						
Akaike (AIC)	113719.9	113262.8	112809.3	112522.4	112273.6	112131.9
Bayesian (BIC)	113834.9	113404.8	112978.4	112718.5	112496.8	112382.1
Entropy	0.824	0.799	0.789	0.787	0.793	0.780
BLRT *p*	**<0.001**	**<0.001**	**<0.001**	**<0.001**	**<0.001**	**<0.001**
Smallest class (%)	**20.3%**	**11.5%**	**7.7%**	4.1%	3.6%	2.1%
**Anxiety models**						
Akaike (AIC)	100683.3	100474.5	100147.8	100010.2	99918.44	99786.97
Bayesian (BIC)	100798.2	100616.5	100316.8	100206.3	100141.6	100037.1
Entropy	0.747	0.738	0.717	0.709	0.704	0.713
BLRT *p*	**<0.001**	**<0.001**	**<0.001**	**<0.001**	**<0.001**	**<0.001**
Smallest class (%)	**25.0%**	**7.3%**	**6.9%**	3.1%	2.9%	2.1%

*Note*: *k*_classes_: number of classes included in the growth mixture model; AIC, Akaike Information Criteria; BIC, Bayesian Information Criteria; BLRT p: *p*-value from Bayesian likelihood ratio test; **bold** values indicate *p* < 0.01 or smallest class size representing >5% of the total sample.

The multinomial regression analyses for depression and anxiety symptom trajectories are summarized in Tables 3 and 4 respectively. The low symptom class was the reference group in the primary analyses, but as specified, the analyses were re-estimated with the high class as the reference group to examine factors that differentiated those who continued to experience high symptoms from those whose symptoms decreased over the follow-up period. In the depression class regression, males had significantly higher odds of being in the low symptom depression class compared to the other three classes (ORs = 0.319–0.767), while gender diverse participants had greater odds of being in the high (OR = 2.269) or decreasing (OR = 1.848) classes compared to females. In terms of diatheses, higher neuroticism was consistently associated with having a poorer trajectory (ORs = 1.188–1.428). Adverse childhood experiences were associated with the high (OR = 1.357) or decreasing (OR = 1.276) trajectory, as were lower levels of conscientiousness (ORs = 0.750, 0.771). Lower levels of socioeconomic disadvantage were significantly associated with lower odds of being in the decreasing class compared to the low symptom class (OR = 0.935). Among the stressor variables, peer problems (ORs = 1.160–1.330) and negative family interactions (ORs = 1.240–2.142) were consistently associated with a high, increasing, or decreasing trajectory, while being bullied was associated with the high (OR = 1.210) or decreasing (OR = 1.123) trajectory. Few variables differentiated between the decreasing and the high classes in the post-hoc analysis, with only male gender (OR = 1.877) and greater socioeconomic disadvantage (OR = 0.920) associated with greater odds of a decreasing trajectory compared to a high trajectory. Factors associated with the anxiety class were identical to those for the depression class, except that gender diverse was not significantly associated with class membership, and negative family interactions were not significantly associated with the increasing class (remaining significant for decreasing and high classes compared to low).

**Table 3.** Regression model of stress–diathesis factors associated with trajectories of depression symptoms (*n* = 6102)

Depression class	Increasing vs. low (1 vs. 4)		Decreasing vs. low (2 vs. 4)		High vs. low (3 vs. 4)		Decreasing vs. high (2 vs. 3)	
	OR (95% CI)	*p*	OR (95% CI)	*p*	OR (95% CI)	*p*	OR (95% CI)	*p*
Intercept		0.310		<0.001		<0.001		0.274
Age	0.858 (0.722, 1.020)	0.082	1.049 (0.879, 1.253)	0.597	0.994 (0.839, 1.177)	0.942	1.056 (0.859, 1.297)	0.606
Gender								
Male vs. female	0.319 (0.251, 0.406)	**<0.001**	0.767 (0.611, 0.962)	0.022	0.409 (0.324, 0.516)	**<0.001**	1.877 (1.416, 2.487)	**<0.001**
Gender diverse vs. female	1.231 (0.776, 1.951)	0.377	1.848 (1.217, 2.806)	**0.004**	2.269 (1.572, 3.275)	**<0.001**	0.814 (0.554, 1.197)	0.296
English vs. another language	1.095 (0.722, 1.659)	0.669	1.009 (0.675, 1.509)	0.963	0.913 (0.618, 1.350)	0.648	1.106 (0.692, 1.768)	0.675
Diatheses								
ICSEA socioeconomic status	1.000 (0.999, 1.002)	0.812	1.002 (1.000, 1.004)	0.019	1.000 (0.998, 1.001)	0.673	1.002 (1.000, 1.004)	0.019
IRSD decile	1.006 (0.963, 1.052)	0.781	0.935 (0.894, 0.978)	**0.003**	1.016 (0.972, 1.063)	0.478	0.920 (0.872, 0.970)	**0.002**
Agreeableness	1.016 (0.952, 1.085)	0.623	0.967 (0.907, 1.032)	0.316	0.999 (0.940, 1.063)	0.984	0.968 (0.901, 1.040)	0.375
Conscientiousness	0.949 (0.894, 1.008)	0.087	0.771 (0.723, 0.821)	**<0.001**	0.750 (0.705, 0.797)	**<0.001**	1.028 (0.955, 1.106)	0.470
Extroversion	0.982 (0.932, 1.035)	0.496	0.982 (0.930, 1.036)	0.502	0.957 (0.910, 1.008)	0.095	1.025 (0.964, 1.090)	0.423
Neuroticism	1.188 (1.124, 1.257)	**<0.001**	1.355 (1.276, 1.440)	**<0.001**	1.428 (1.347, 1.514)	**<0.001**	0.949 (0.884, 1.019)	0.152
Openness to experience	0.972 (0.910, 1.038)	0.399	1.006 (0.941, 1.075)	0.869	1.019 (0.956, 1.086)	0.563	0.987 (0.915, 1.064)	0.732
Adverse childhood events	1.046 (0.967, 1.131)	0.263	1.276 (1.192, 1.367)	**<0.001**	1.357 (1.273, 1.448)	**<0.001**	0.940 (0.873, 1.013)	0.105
Disability present vs. absent	1.167 (0.866, 1.573)	0.311	1.338 (1.010, 1.771)	0.042	1.339 (1.023, 1.751)	0.033	0.999 (0.732, 1.363)	0.996
Stressors								
Peer problems	1.160 (1.086, 1.238)	**<0.001**	1.260 (1.182, 1.344)	**<0.001**	1.330 (1.252, 1.413)	**<0.001**	0.947 (0.883, 1.017)	0.134
Bullied	1.026 (0.950, 1.108)	0.511	1.123 (1.050, 1.201)	**<0.001**	1.210 (1.139, 1.286)	**<0.001**	0.928 (0.867, 0.994)	0.032
Negative friend interactions	1.008 (0.862, 1.178)	0.925	0.906 (0.775, 1.059)	0.215	0.861 (0.743, 0.998)	0.047	1.052 (0.883, 1.255)	0.569
Negative family interactions	1.240 (1.081, 1.422)	**0.002**	1.988 (1.728, 2.287)	**<0.001**	2.142 (1.876, 2.447)	**<0.001**	0.928 (0.790, 1.090)	0.361

*Note*: **bold** values indicate *p* < 0.01; ICSEA, Index of Community Socio-Educational Advantage; IRSD, Index of Relative Socio-economic Disadvantage.

**Table 4.** Regression model of stress–diathesis factors associated with trajectories of anxiety symptoms (*n* = 6102)

Anxiety class	Increasing vs. low (1 vs. 4)		Decreasing vs. low (2 vs. 4)		High vs. low (3 vs. 4)		Decreasing vs. high (2 vs. 3)	
	OR (95% CI)	*p*	OR (95% CI)	*p*	OR (95% CI)	*p*	OR (95% CI)	*p*
Intercept		0.176		<0.001		<0.001		0.479
Age	0.859 (0.723, 1.020)	0.083	1.026 (0.845, 1.245)	0.798	0.995 (0.858, 1.153)	0.942	1.031 (0.843, 1.261)	0.764
Gender								
Male vs. female	0.200 (0.157, 0.255)	**<0.001**	0.441 (0.344, 0.564)	**<0.001**	0.151 (0.122, 0.188)	**<0.001**	2.915 (2.202, 3.859)	**<0.001**
Gender diverse vs. female	0.860 (0.528, 1.401)	0.545	1.181 (0.755, 1.847)	0.466	1.018 (0.712, 1.456)	0.920	1.160 (0.777, 1.730)	0.468
English vs. another language	1.362 (0.876, 2.118)	0.170	0.935 (0.613, 1.424)	0.753	1.363 (0.940, 1.977)	0.102	0.686 (0.433, 1.085)	0.107
Diatheses								
ICSEA socioeconomic status	1.000 (0.998, 1.001)	0.621	1.001 (1.000, 1.003)	0.097	1.000 (0.998, 1.001)	0.592	1.002 (1.000, 1.004)	0.046
IRSD decile	1.054 (1.007, 1.103)	0.024	0.920 (0.877, 0.966)	**<0.001**	1.011 (0.972, 1.051)	0.582	0.910 (0.865, 0.957)	**<0.001**
Agreeableness	1.053 (0.985, 1.126)	0.130	0.995 (0.928, 1.067)	0.886	1.054 (0.997, 1.114)	0.062	0.944 (0.880, 1.012)	0.106
Conscientiousness	1.002 (0.943, 1.064)	0.955	0.863 (0.807, 0.924)	**<0.001**	0.895 (0.850, 0.943)	**<0.001**	0.965 (0.900, 1.034)	0.308
Extroversion	0.997 (0.946, 1.052)	0.922	0.959 (0.905, 1.017)	0.162	0.970 (0.928, 1.015)	0.187	0.989 (0.932, 1.049)	0.707
Neuroticism	1.228 (1.159, 1.301)	**<0.001**	1.771 (1.651, 1.901)	**<0.001**	1.936 (1.831, 2.047)	**<0.001**	0.915 (0.850, 0.985)	0.019
Openness to experience	1.034 (0.968, 1.105)	0.324	1.087 (1.012, 1.168)	0.023	1.063 (1.005, 1.125)	0.033	1.022 (0.950, 1.100)	0.556
Adverse childhood events	0.996 (0.918, 1.082)	0.931	1.226 (1.137, 1.322)	**<0.001**	1.198 (1.127, 1.274)	**<0.001**	1.023 (0.949, 1.104)	0.549
Disability present vs. absent	0.829 (0.592, 1.163)	0.277	1.327 (0.979, 1.798)	0.068	1.242 (0.969, 1.594)	0.088	1.068 (0.784, 1.453)	0.677
Stressors								
Peer problems	1.120 (1.046, 1.199)	**0.001**	1.184 (1.104, 1.269)	**<0.001**	1.233 (1.167, 1.303)	**<0.001**	0.960 (0.895, 1.030)	0.253
Bullied	1.043 (0.961, 1.132)	0.314	1.138 (1.057, 1.225)	**<0.001**	1.199 (1.129, 1.272)	**<0.001**	0.950 (0.885, 1.018)	0.147
Negative friend interactions	1.028 (0.875, 1.207)	0.741	1.228 (1.038, 1.452)	0.017	1.159 (1.016, 1.323)	0.028	1.059 (0.893, 1.256)	0.511
Negative family interactions	1.134 (0.987, 1.304)	0.077	1.400 (1.204, 1.627)	**<0.001**	1.391 (1.239, 1.563)	**<0.001**	1.006 (0.862, 1.174)	0.939

*Note*: **bold** values indicate *p* < 0.01; ICSEA, Index of Community Socio-educational Advantage; IRSD, Index of Relative Socio-economic Disadvantage.

Sensitivity analyses included re-estimating the models accounting for clustering by school, and re-estimating the models with five missing data imputed datasets. There were no differences in significant (and non-significant) factors identified in the mixed effects models that accounted for clustering, compared to the models presented in Tables 3 and 4 (see Supplementary Table S2). Multiple imputation also had minimal effect on the model outcomes, with only the effect of the IRSD decile indicator of socioeconomic status becoming non-significant (from *p* = 0.003 to *p* = 0.014) in the imputed dataset for the comparison between decreasing and low depression trajectories (see Supplementary Table S3).

## Discussion

The findings of this study demonstrate the utility of using the stress–diathesis model to guide a longitudinal investigation to identify adolescents at risk of poor trajectories of depression and anxiety, and the factors increasing these trajectory profiles. Several diatheses, or background vulnerabilities, were significantly associated with poorer trajectories. After accounting for significant gender differences, these included lower socioeconomic status, greater exposure to adverse childhood experiences, and higher neuroticism and lower conscientiousness. Similarly, specific stressors substantively influenced trajectories of depression and anxiety symptoms, particularly experiences of peer problems and bullying, and negative interactions with family.

Across both depression and anxiety trajectories, four distinct classes were identified, with approximately two-thirds of participants exhibiting low and stable symptoms across the follow-up period and another 6–7% with decreasing symptoms. Of concern are the remaining 19–26% of participants who consistently reported a high level of symptoms or an increasing symptom trajectory for depression (19.2%) and anxiety (26.2%). That approximately a quarter of our sample showed high-risk trajectories is consistent with the broader literature indicating high levels of depression and anxiety among young people (Australian Bureau of Statistics, 2023a; Lu, Lin, & Su, 2024).

The extracted trajectory classes were largely consistent for depression and anxiety. The four distinct classes identified for the depression trajectories are consistent with the broader literature in which a four-class solution has most frequently been identified (Shore et al., 2018). The four anxiety trajectory classes differ from previous studies, which have typically identified two, three, or five trajectory classes (Crocetti et al., 2009; Morin et al., 2011; Salto et al., 2025; Spence et al., 2022). It is likely that the differing findings reflect methodological differences (including sample size, age of participants, and timing of assessments).

Our study extends the literature by examining depression and anxiety trajectories in the same sample and found that the depression class was only moderately associated with the anxiety class. This finding is consistent with evidence showing moderate levels of comorbidity between anxiety and depression during childhood and adolescence (Cummings, Caporino, & Kendall, 2014; Pigatto et al., 2025) and suggests that, despite some comorbidity in our sample, anxiety symptoms did not always track alongside depression symptoms for individuals within the cohort. Factors influencing depression and anxiety trajectories were largely common, which may reflect similar underlying vulnerabilities. This is consistent with recent findings showing that risk for comorbidity cumulatively increases as exposure to more common risk factors increases (Pigatto et al., 2025), including factors measured in our study (e.g. bullying, ACEs). The role of comorbidity could be tested in future studies by examining co-occurring trajectories of depression and anxiety, and predictors of these trajectories. Based on our findings of shared risk factors in our study, preventive approaches targeting these factors may have value in reducing the likelihood of high-risk depression and anxiety trajectories.

These findings suggest that preventative approaches need to be multidimensional, addressing both modifiable and non-modifiable factors. Specifically, our work found that females and gender diverse adolescents experience poorer trajectories, which fits with a well-established literature indicating that these groups experience worse mental health (e.g. Salk, Hyde, & Abramson, 2017; Smout et al., 2025; Su et al., 2024). Future research that adopts an understanding of the inequitable effects of gender on mental health and applies this gender lens to the development of initiatives to address this inequity is needed.

Our study suggests that programs aiming to reduce the incidence of adverse childhood experiences should continue to be promoted and expanded, given their wide-ranging impact (Grummitt et al., 2024; Hughes et al., 2017). Commonly reported adverse childhood experiences in our sample included verbal abuse from parent/s, living with someone experiencing a mental illness or someone who was an alcoholic, experiencing a significant threat of harm, and/or having parents who had separated or divorced. Programs may be able to address at least some of these factors, including expanding perinatal and parenting programs (inclusive of parenting programs for diverse genders), providing affordable childcare, and providing services that reduce family violence and alcohol and substance abuse. Accessible mental health and well-being services in community settings, including youth-friendly clinics, school-based counselling, and digital supports, are also critical for reducing barriers to early help-seeking and ensuring timely intervention.

Within schools, greater investment in preventing peer problems and bullying should be prioritized. School staff require training in social–emotional skills programs and small group social skill supports with appropriate resources to ensure that these programs are implemented effectively. While evidence-based programs exist (Gaffney, Ttofi, & Farrington, 2021), implementation barriers such as limited leadership support, low staff engagement, and insufficient resourcing often constrain delivery in schools (Moore et al., 2022). Schools also provide an environment in which large groups of young people can be readily screened for symptoms and connected with services and support, which ideally might disrupt a pathway towards a poorer symptom trajectory over time. The identification of distinct high and increasing symptom trajectories also suggests that it would be valuable to monitor change over time. Several studies have examined the effects of delivering school-based screening, with one study showing that web-based screening for depression and anxiety led to increased help-seeking intentions (O’Dea et al., 2021), while another found that school-based screening involving feedback to caregivers improved student mental health a year later, but had no effect on service use (Rapee et al., 2025). This approach shows some promise, but will only work if there are effective programs and services available to support the young people identified by screening who require support. Greater funding from governments for mental health services and close collaboration between the education and health sectors are likely required for this promise to be realized.

Finally, strengthening the family environment may also mitigate the risk of poorer trajectories, highlighting the importance of accessible parent resources and evidence-based family skills programs, that are inclusive of diverse genders. Approaches that target conflict resolution, communication, and boundary-setting during adolescence, combined with increased financial and social support for families, are likely to be beneficial (Kuhn & Laird, 2014).

### Strengths and limitations

The current study is one of the first to characterize trajectories of depression and anxiety symptoms in a large population-based national cohort of adolescents. A theoretical framework was used to select candidate risk factors for the models, with a wide selection of potential factors measured within the cohort. The GMM approach used all available data, adopting realistic assumptions around missingness. Sensitivity analyses accounted for clustering, and the use of multiple imputation to account for missing data made a negligible difference to the outcomes, suggesting that findings were robust. Findings provide guidance for improving supports and services available to adolescents in Australia.

Nevertheless, several limitations are acknowledged. Assessments were conducted every year, precluding examination of shorter-term trajectories. The measures, while well-validated, were based on self-report, as clinical validation was not feasible in the context of a large population-based cohort study. Some of the independent variables had suboptimal psychometric properties, particularly the SDQ, while two-item indices of core personality factors may be unlikely to provide a rich assessment of vulnerability. Nevertheless, some of these variables had significant associations with poorer trajectories. There is also a range of additional stressors and diatheses that were not feasible to measure but would merit attention in future research (e.g. other adverse childhood experiences such as abuse). Relatedly, some of the assessed diatheses may reflect or overlap with stressors, such as ongoing adverse events or experiences of disadvantage. Further, we did not provide a direct test of the stress–diathesis model, which would require the inclusion of a stress by diathesis interaction term, which could then be used as a predictor of class membership. Testing the stress diathesis model in future studies would provide insight into the applicability of the model to predict high-risk trajectories over time during adolescence. Attrition was between 24–45% at the follow-up assessments, which is not unusual in large school-based cohorts, and primarily reflected schools not engaging in assessments rather than individual students dropping out. Finally, GMM tends to support a similar pattern of trajectories across diverse data, representing low, increasing, decreasing, and chronic symptoms. It has been suggested that this pattern may partly reflect a modelling artifact rather than a true reflection of distinct subgroups (Sher, Jackson, & Steinley, 2011), so investigation of alternative trajectory modelling methods, such as latent growth models to characterize individual trajectories or joint modelling of depression and anxiety trajectories, may be warranted.

The present study contributes to the literature by examining depression and anxiety trajectories during adolescence and the predictors of these trajectories as informed by the stress–diathesis model. Findings identified four distinct trajectories of depression and anxiety symptoms across 4 years at a developmentally critical stage. The trajectories included consistently low symptoms, consistently high symptoms, elevated symptoms that reduced over time, and low-moderate symptoms that increased over time. Approximately a fifth to a quarter of the sample were in the higher risk (high, increasing) trajectories, and predictors of these trajectories were being female or gender diverse, lower socioeconomic status, higher levels of neuroticism and lower levels of conscientiousness, greater adverse childhood experiences, higher levels of peer problems, bullying victimization, and negative family interactions. These findings highlight the need for prevention programs and policy initiatives to focus on these factors, many of which require addressing societal inequities that elevate risk for the development of adolescent mental health problems.

## Supporting information

10.1017/S0033291726103560.sm001 Batterham et al. supplementary material Batterham et al. supplementary material

## Supplementary material

The supplementary material for this article can be found at https://doi.org/10.1017/S0033291726103560.
