# Supplementary material for: Stress-diathesis based predictors of depression and anxiety trajectories in adolescence: a population-based longitudinal cohort study
Source: Psychol Med. 2026 Mar 3;56:e64. doi: 10.1017/S0033291726103560 (PMC12969213; doi:10.1017/S0033291726103560)
Supplement: Batterham et al. supplementary material [file S0033291726103560sup001.docx]

Supplementary Table 1: Zero-order bivariate correlations of the anxiety and depression measures at all timepoints

|  | PHQ-A baseline | PHQ-A  12mo | PHQ-A  24mo | PHQ-A 36mo | SCAS-GAD baseline | SCAS-GAD 12mo | SCAS-GAD 24mo | SCAS-GAD 36mo |
| --- | --- | --- | --- | --- | --- | --- | --- | --- |
| PHQ-A baseline | 1.000 | 0.582 | 0.527 | 0.455 | 0.702 | 0.451 | 0.410 | 0.370 |
| PHQ-A 12mo | 0.582 | 1.000 | 0.661 | 0.574 | 0.506 | 0.701 | 0.532 | 0.485 |
| PHQ-A 24mo | 0.527 | 0.661 | 1.000 | 0.662 | 0.478 | 0.556 | 0.700 | 0.538 |
| PHQ-A 36mo | 0.455 | 0.574 | 0.662 | 1.000 | 0.424 | 0.498 | 0.527 | 0.688 |
| SCAS-GAD baseline | 0.702 | 0.506 | 0.478 | 0.424 | 1.000 | 0.626 | 0.568 | 0.535 |
| SCAS-GAD 12mo | 0.451 | 0.701 | 0.556 | 0.498 | 0.626 | 1.000 | 0.714 | 0.654 |
| SCAS-GAD 24mo | 0.410 | 0.532 | 0.700 | 0.527 | 0.568 | 0.714 | 1.000 | 0.710 |
| SCAS-GAD 36mo | 0.370 | 0.485 | 0.538 | 0.688 | 0.535 | 0.654 | 0.710 | 1.000 |

Supplementary Table 2: Estimates of intercept, linear change and quadratic change from the final growth mixture models

|  | Intercept | | | Linear change | | | Quadratic change | | |
| --- | --- | --- | --- | --- | --- | --- | --- | --- | --- |
|  | Estimate | SE | p | Estimate | SE | p | Estimate | SE | p |
| ***Depression symptom class*** |  |  |  |  |  |  |  |  |  |
| High | 7.553 | 1.507 | **<0.001** | -3.012 | 1.978 | 0.128 | -0.344 | 0.186 | 0.064 |
| Low | 4.468 | 0.102 | **<0.001** | 0.067 | 0.106 | 0.524 | 0.085 | 0.034 | 0.012 |
| Increasing | 7.483 | 0.623 | **<0.001** | 9.511 | 0.667 | **<0.001** | -2.647 | 0.241 | **<0.001** |
| Decreasing | 16.816 | 0.485 | **<0.001** | -11.039 | 0.631 | **<0.001** | 2.745 | 0.186 | **<0.001** |
| ***Anxiety symptom class*** |  |  |  |  |  |  |  |  |  |
| High | 12.337 | 0.232 | **<0.001** | 0.064 | 0.302 | 0.833 | -0.255 | 0.104 | 0.014 |
| Low | 4.186 | 0.074 | **<0.001** | -0.302 | 0.085 | **<0.001** | 0.116 | 0.028 | **<0.001** |
| Increasing | 6.016 | 0.311 | **<0.001** | 5.673 | 0.752 | **<0.001** | -1.505 | 0.302 | **<0.001** |
| Decreasing | 12.332 | 0.251 | **<0.001** | -7.771 | 0.430 | **<0.001** | 1.953 | 0.162 | **<0.001** |

Supplementary Table 3: Summary of regression models re-estimated accounting for clustering by school

|  | Depression symptom class | | | | | |  | Anxiety symptom class | | | | | |
| --- | --- | --- | --- | --- | --- | --- | --- | --- | --- | --- | --- | --- | --- |
|  | Increasing vs low | | Decreasing vs low | | High vs low | |  | Increasing vs low | | Decreasing vs low | | High vs low | |
|  | OR | p | OR | p | OR | p |  | OR | p | OR | p | OR | p |
| Intercept |  | 0.314 |  | <0.001 |  | <0.001 |  |  | 0.154 |  | <0.001 |  | <0.001 |
| Age | 0.858 | 0.085 | 1.049 | 0.600 | 0.994 | 0.942 |  | 0.859 | 0.068 | 1.026 | 0.787 | 0.995 | 0.939 |
| Gender |  |  |  |  |  |  |  |  |  |  |  |  |  |
| Male vs female | 0.319 | **<0.001** | 0.767 | 0.023 | 0.409 | **<0.001** |  | 0.200 | **<0.001** | 0.441 | **<0.001** | 0.151 | **<0.001** |
| Gender diverse vs female | 1.231 | 0.381 | 1.848 | **0.004** | 2.269 | **<0.001** |  | 0.860 | 0.523 | 1.181 | 0.442 | 1.018 | 0.916 |
| English vs another language | 1.095 | 0.672 | 1.009 | 0.964 | 0.913 | 0.651 |  | 1.362 | 0.148 | 0.935 | 0.740 | 1.363 | 0.085 |
| ***Diatheses*** | |  |  |  |  |  |  |  |  |  |  |  |  |
| ICSEA socioeconomic status | 1.000 | 0.813 | 1.002 | 0.020 | 1.000 | 0.675 |  | 1.000 | 0.602 | 1.001 | 0.080 | 1.000 | 0.572 |
| IRSD decile | 1.006 | 0.783 | 0.935 | **0.004** | 1.016 | 0.482 |  | 1.054 | 0.017 | 0.920 | **<0.001** | 1.011 | 0.562 |
| Agreeableness | 1.016 | 0.626 | 0.967 | 0.320 | 0.999 | 0.984 |  | 1.053 | 0.110 | 0.995 | 0.880 | 1.054 | 0.050 |
| Conscientiousness | 0.949 | 0.090 | 0.771 | **<0.001** | 0.750 | **<0.001** |  | 1.002 | 0.953 | 0.863 | **<0.001** | 0.895 | **<0.001** |
| Extroversion | 0.982 | 0.499 | 0.982 | 0.506 | 0.957 | 0.098 |  | 0.997 | 0.918 | 0.959 | 0.141 | 0.970 | 0.165 |
| Neuroticism | 1.188 | **<0.001** | 1.355 | **<0.001** | 1.428 | **<0.001** |  | 1.228 | **<0.001** | 1.771 | **<0.001** | 1.936 | **<0.001** |
| Openness to experience | 0.972 | 0.403 | 1.006 | 0.870 | 1.019 | 0.566 |  | 1.034 | 0.298 | 1.087 | 0.016 | 1.063 | 0.024 |
| Adverse childhood events | 1.046 | 0.267 | 1.276 | **<0.001** | 1.357 | **<0.001** |  | 0.996 | 0.928 | 1.226 | **<0.001** | 1.198 | **<0.001** |
| Disability present vs absent | 1.167 | 0.315 | 1.338 | 0.044 | 1.339 | 0.035 |  | 0.829 | 0.252 | 1.327 | 0.055 | 1.242 | 0.072 |
| ***Stressors*** | |  |  |  |  |  |  |  |  |  |  |  |  |
| Peer problems | 1.160 | **<0.001** | 1.260 | **<0.001** | 1.330 | **<0.001** |  | 1.120 | **<0.001** | 1.184 | **<0.001** | 1.233 | **<0.001** |
| Bullied | 1.026 | 0.515 | 1.123 | **<0.001** | 1.210 | **<0.001** |  | 1.043 | 0.288 | 1.138 | **<0.001** | 1.199 | **<0.001** |
| Negative friend interactions | 1.008 | 0.925 | 0.906 | 0.219 | 0.861 | 0.049 |  | 1.028 | 0.727 | 1.228 | 0.012 | 1.159 | 0.020 |
| Negative family interactions | 1.240 | **0.002** | 1.988 | **<0.001** | 2.142 | **<0.001** |  | 1.134 | 0.062 | 1.400 | **<0.001** | 1.391 | **<0.001** |

*Notes*: **bold** values indicate *p* < .01; ICSEA: Index of Community Socio-Educational Advantage; IRSD: Index of Relative Socio-economic Disadvantage

Supplementary Table 4: Summary of regression models re-estimated accounting for missing data using pooled data from five multiple imputations

|  | Depression symptom class | | | | | |  | Anxiety symptom class | | | | | |
| --- | --- | --- | --- | --- | --- | --- | --- | --- | --- | --- | --- | --- | --- |
|  | Increasing vs low | | Decreasing vs low | | High vs low | |  | Increasing vs low | | Decreasing vs low | | High vs low | |
|  | OR | p | OR | p | OR | p |  | OR | p | OR | p | OR | p |
| Intercept |  | 0.390 |  | <0.001 |  | <0.001 |  |  | 0.150 |  | <0.001 |  | <0.001 |
| Age | 0.848 | 0.057 | 1.046 | 0.610 | 0.977 | 0.787 |  | 0.850 | 0.061 | 1.055 | 0.579 | 1.026 | 0.728 |
| Gender |  |  |  |  |  |  |  |  |  |  |  |  |  |
| Male vs female | 0.323 | **<0.001** | 0.751 | 0.011 | 0.399 | **<0.001** |  | 0.200 | **<0.001** | 0.445 | **<0.001** | 0.159 | **<0.001** |
| Gender diverse vs female | 1.311 | 0.230 | 1.805 | **0.005** | 2.176 | **<0.001** |  | 0.844 | 0.487 | 1.133 | 0.579 | 1.003 | 0.985 |
| English vs another language | 1.039 | 0.852 | 0.996 | 0.983 | 0.953 | 0.808 |  | 1.388 | 0.144 | 0.963 | 0.859 | 1.374 | 0.090 |
| ***Diatheses*** |  |  |  |  |  |  |  |  |  |  |  |  |  |
| ICSEA socioeconomic status | 1.000 | 0.860 | 1.002 | 0.024 | 1.000 | 0.655 |  | 1.000 | 0.797 | 1.002 | 0.079 | 1.000 | 0.603 |
| IRSD decile | 1.005 | 0.831 | 0.946 | 0.014 | 1.021 | 0.357 |  | 1.051 | 0.029 | 0.925 | **0.001** | 1.015 | 0.439 |
| Agreeableness | 1.016 | 0.639 | 0.971 | 0.371 | 0.999 | 0.965 |  | 1.051 | 0.147 | 0.998 | 0.960 | 1.060 | 0.039 |
| Conscientiousness | 0.950 | 0.108 | 0.772 | **<0.001** | 0.755 | **<0.001** |  | 1.005 | 0.867 | 0.862 | **<0.001** | 0.895 | **<0.001** |
| Extroversion | 0.983 | 0.520 | 0.981 | 0.492 | 0.953 | 0.065 |  | 0.996 | 0.872 | 0.961 | 0.184 | 0.972 | 0.217 |
| Neuroticism | 1.193 | **<0.001** | 1.344 | **<0.001** | 1.409 | **<0.001** |  | 1.227 | **<0.001** | 1.760 | **<0.001** | 1.929 | **<0.001** |
| Openness to experience | 0.969 | 0.339 | 1.003 | 0.934 | 1.021 | 0.514 |  | 1.034 | 0.320 | 1.090 | 0.022 | 1.068 | 0.026 |
| Adverse childhood events | 1.052 | 0.200 | 1.256 | **<0.001** | 1.338 | **<0.001** |  | 1.000 | 0.995 | 1.217 | **<0.001** | 1.195 | **<0.001** |
| Disability present vs absent | 1.167 | 0.300 | 1.409 | 0.013 | 1.347 | 0.025 |  | 0.892 | 0.489 | 1.336 | 0.057 | 1.257 | 0.063 |
| ***Stressors*** |  |  |  |  |  |  |  |  |  |  |  |  |  |
| Peer problems | 1.150 | **<0.001** | 1.253 | **<0.001** | 1.328 | **<0.001** |  | 1.117 | **0.001** | 1.174 | **<0.001** | 1.231 | **<0.001** |
| Bullied | 1.029 | 0.460 | 1.127 | **<0.001** | 1.220 | **<0.001** |  | 1.053 | 0.214 | 1.139 | **<0.001** | 1.198 | **<0.001** |
| Negative friend interactions | 1.015 | 0.850 | 0.906 | 0.213 | 0.862 | 0.048 |  | 1.028 | 0.734 | 1.221 | 0.018 | 1.152 | 0.032 |
| Negative family interactions | 1.238 | **0.002** | 1.985 | **<0.001** | 2.126 | **<0.001** |  | 1.135 | 0.073 | 1.394 | **<0.001** | 1.397 | **<0.001** |

*Notes*: **bold** values indicate *p* < .01; ICSEA: Index of Community Socio-Educational Advantage; IRSD: Index of Relative Socio-economic Disadvantage

**Supplementary Materials: Annotated code used to conduct analyses**

**Mplus code for developing growth mixture models**

TITLE: GMM on GAD symptoms (repeated with depression symptoms)

DATA: FILE IS 'C:\Documents\fp36.dat';

VARIABLE: NAMES ARE id dq1 dq2 dq3 dq4

dep1 dep2 dep3 dep4 si1 si2 si3 si4

gad1 gad2 gad3 gad4 sp1 sp2 sp3 sp4;

MISSING ARE all (-999);

*!*** (Vary number of classes from 2-7).*

CLASSES = c (4);

USEVARIABLES ARE gad1 gad2 gad3 gad4;

ANALYSIS:

TYPE = MIXTURE;

STARTS = 600 20;

LRTSTARTS = 0 0 300 10;

MODEL:

%OVERALL%

i s q | gad1@0 gad2@1 gad3@2 gad4@3;

OUTPUT: TECH10 TECH14;

*!*** Save the estimated most probable class for each participant.*

Savedata:

file is 'C:\Documents\gadclass.dat';

save is cprob;

format is free;

**SPSS code for describing class membership and testing correlates of class membership**

**** Open merged dataset with baseline predictors and class membership indicators.*

GET FILE="C:\Documents\Future proofing\fpclass.sav".

**** Align GAD trajectory classes with depression trajectory classes (high with high etc).*

RECODE gadclass (4=1)(3=2)(1=3)(2=4) INTO gadclassr.

**** Comparison of depression and anxiety classes (depclass, gadclassr), including Spearman correlation.*

CROSSTAB depclass by gadclassr /stat=chisq /cell=col row count.

NONPAR CORR /VARIABLES=depclass gadclassr /PRINT=SPEARMAN TWOTAIL NOSIG FULL /MISSING=PAIRWISE.

**** Characterising trajectories based on mean PHQ-9/GAD-7 scores for each latent class.*

MEAN PHQ9_Total_Score PHQ9_Total_Score_M12 PHQ9_Total_Score_M24 PHQ9_Total_Score_M36 SCAS_GAD_Subscale SCAS_GAD_Subscale_M12 SCAS_GAD_Subscale_M24 SCAS_GAD_Subscale_M36 by depclass gadclass.

**** Diathesis variables: Agreeableness Conscientiousness Extroversion Neuroticism Openness_to_Experience ICSEA IRSD_Decile disability ACE.*

**** Stressor variables: Peer_problems BULY1 BULY2 BULY3 SSSS_Friends_Negative SSSS_Family_Negative.*

**** Potential demographic confounds: CALD1 GNDR2 age.*

****** Multinominal regressions to identify stress-diathesis correlates of class membership.*

NOMREG depclass (BASE=LAST ORDER=ASCENDING) BY GNDR2x englishx disability WITH Age_At_Entry ICSEA IRSD_Decile Agreeableness Conscientiousness Extroversion Neuroticism Openness_to_Experience Peer_problems bullied acetot SSSS_Friends_Negative SSSS_Family_Negative /MODEL /INTERCEPT=INCLUDE /PRINT=PARAMETER SUMMARY LRT CPS STEP MFI.

NOMREG gadclassr (BASE=LAST ORDER=ASCENDING) BY GNDR2x englishx disability WITH Age_At_Entry ICSEA IRSD_Decile Agreeableness Conscientiousness Extroversion Neuroticism Openness_to_Experience Peer_problems bullied acetot SSSS_Friends_Negative SSSS_Family_Negative /MODEL /INTERCEPT=INCLUDE /PRINT=PARAMETER SUMMARY LRT CPS STEP MFI.

******* Sensitivity analyses.*

**** Sensitivity analysis 1: Accounting for clustering (generalised mixed effects models).*

GENLINMIXED /DATA_STRUCTURE SUBJECTS=user_id*School_Id /FIELDS TARGET=depclass /TARGET_OPTIONS DISTRIBUTION=MULTINOMIAL LINK=LOGIT /FIXED EFFECTS=GNDR2x englishx disability Age_At_Entry ICSEA IRSD_Decile Agreeableness Conscientiousness Extroversion Neuroticism Openness_to_Experience Peer_problems bullied acetot SSSS_Friends_Negative SSSS_Family_Negative USE_INTERCEPT=TRUE.

GENLINMIXED /DATA_STRUCTURE SUBJECTS=user_id*School_Id /FIELDS TARGET=gadclassr /TARGET_OPTIONS DISTRIBUTION=MULTINOMIAL LINK=LOGIT /FIXED EFFECTS=GNDR2x englishx disability Age_At_Entry ICSEA IRSD_Decile Agreeableness Conscientiousness Extroversion Neuroticism Openness_to_Experience Peer_problems bullied acetot SSSS_Friends_Negative SSSS_Family_Negative USE_INTERCEPT=TRUE.

**** Sensitivity analysis 2: Accounting for missing predictors using multiple imputation and re-estimating models using pooled estimates from 5 imputed datasets.*

VARIABLE LEVEL IRSD_Decile Agreeableness Conscientiousness Extroversion Neuroticism Openness_to_Experience Peer_problems bullied (scale).

MULTIPLE IMPUTATION GNDR2x englishx disability Age_At_Entry ICSEA IRSD_Decile Agreeableness Conscientiousness Extroversion Neuroticism Openness_to_Experience Peer_problems bullied acetot SSSS_Friends_Negative SSSS_Family_Negative depclass gadclassr /IMPUTE METHOD=AUTO MAXITER= 10 MAXMODELPARAM=200 NIMPUTATIONS=5 SCALEMODEL=LINEAR INTERACTIONS=NONE SINGULAR=1E-012 MAXPCTMISSING=NONE /MISSINGSUMMARIES NONE /IMPUTATIONSUMMARIES MODELS DESCRIPTIVES /OUTFILE IMPUTATIONS="C:\Documents\Future proofing\fpclassmi.sav".

GET FILE="C:\Documents\Future proofing\fpclassmi.sav".

SPLIT FILE by imputation_.

NOMREG depclass (BASE=LAST ORDER=ASCENDING) BY GNDR2x englishx disability WITH Age_At_Entry ICSEA IRSD_Decile Agreeableness Conscientiousness Extroversion Neuroticism Openness_to_Experience Peer_problems bullied acetot SSSS_Friends_Negative SSSS_Family_Negative /MODEL /INTERCEPT=INCLUDE /PRINT=PARAMETER SUMMARY LRT CPS STEP MFI.

NOMREG gadclassr (BASE=LAST ORDER=ASCENDING) BY GNDR2x englishx disability WITH Age_At_Entry ICSEA IRSD_Decile Agreeableness Conscientiousness Extroversion Neuroticism Openness_to_Experience Peer_problems bullied acetot SSSS_Friends_Negative SSSS_Family_Negative /MODEL /INTERCEPT=INCLUDE /PRINT=PARAMETER SUMMARY LRT CPS STEP MFI.
